# Characteristic and phylogenetic analyses of chloroplast genome for *Ephedra monosperma* (Ephedraceae), an important medicinal species

**DOI:** 10.1080/23802359.2021.1872440

**Published:** 2021-02-09

**Authors:** Yan-Ling Zhao, Yong-Jun Fan, Yan-Ci Yang

**Affiliations:** School of Biological Science and Technology, Baotou Teachers’ College, Baotou, China

**Keywords:** *Ephedra monosperma*, chloroplast genome, phylogenetic analysis

## Abstract

*Ephedra monosperma* is an important medicinal plant of *Ephedra* (Ephedraceae). The complete chloroplast genome of *E. monosperma* was assembled from Illumina pair-end sequence reads. The whole chloroplast (cp) genome is 109,548 bp in length and presents a quadripartite structure consisting of two copies of inverted repeat (IR) regions (20,398) separated by a large single copy (LSC) region (60,674 bp) and a small single copy (SSC) region (8078 bp). The cp genome of *E. monosperma* encodes a total of 118 genes, including 73 protein-coding genes, 37 tRNA genes and 8 rRNA genes. The overall GC content of *E. monosperma* cp genome is 36.6%. A maximum likelihood (ML) phylogenetic analysis revealed that *E. monosperma* was close to *Ephedra equisetina*. The ML tree also showed Ephedraceae appeared more closely related to Gnetaceae than to the other families in Gymnospermae.

*Ephedra* plants have been used as medicines for several thousand years (Editorial Committee of Zhonghua Bencao National Traditional Chinese Herb Administration 1996; Caveney et al. 2001). *Ephedra monosperma* Gmel. ex C.A. Mey., a drought-tolerant shrub, has high content of ephedrine and pseudoephedrine (Ibragic and Sofić 2015). To date, there have been few studies for this medicinally and ecologically important plant (e.g. Sofronova et al. 2014；Ibragic and Sofić 2015). In this study, we report and characterize the complete chloroplast (cp) genome of *E. monosperma* (GenBank accession number: MW186779) to provide genomic data for future research.

An individual of *E. monosperma* was collected in Chunkun Mountain, Baotou, Inner Mongolia, China (E110°44′, N41°17′). The specimen was deposited at the herbarium of Baotou Teachers’ College (email: yycjyl@163.com) under the voucher number CKS-2020-07-B07DZMH. Total genomic DNA was isolated from silica-dried leaf material using modified CTAB method (Doyle 1987), and then sequenced using an Illumina Hiseq 2500 platform at Biomarker Technologies Inc. (Beijing, China). A total of 574,210 reads were assembled to generate the cp genome of *E. monosperma*. Reference-guided assembly was used to reconstruct the cp genomes with the programs MIRA 4.0.2 (Chevreux et al. 2004) and MITObim v1.7 (Hahn et al. 2013). In the process, cp genome of *Ephedra intermedia* (NC_044772) was used as reference genome. The complete cp genome was annotated in GENEIOUS R10 (Biomatters Ltd., Auckland, New Zealand), and then manually corrected by comparing with the published complete cp genomes (GenBank accession numbers: NC_011954, NC_044772) of *Ephedra* species using GENEIOUS R10.

The complete cp genome of *E. monosperma* is 109,548 bp in length with a quadripartite structure. The large single copy (LSC) region, small single copy (SSC) region and inverted repeat (IRa and IRb) regions are 60,674 bp, 8078 bp and 20,398 bp, respectively. The assembled cp genome encodes a total of 118 genes, consisting of 73 protein-coding genes, 37 tRNA genes, and 8 rRNA genes. The overall GC content of *E. monosperma* cp genome is 36.6%, and the corresponding values in LSC, SSC and IR regions are 34.2%, 27.4%, and 42.1%, respectively.

The phylogenetic tree was constructed based on 17 complete cp genomes of vascular plants. All of the 17 complete cp genome sequences were aligned using MAFFT (Katoh and Standley 2013) with default parameter. To reconstruct the phylogenetic relations of these species we used the GTR + G model with maximum likelihood (ML) analysis. A ML tree was constructed with RAxML v8 (Stamatakis 2014). The node stability was assessed using a rapid bootstrapping analysis with 1000 replicates. The phylogenetic tree indicated that *E. monosperma* was close to *Ephedra equisetina* with high support (Figure 1). The ML tree also showed Ephedraceae appeared more closely related to Gnetaceae than to the other families in Gymnospermae, which is consistent with previous molecular results (Chen et al. 2019). The cp genome of *E. monosperma* will provide useful genetic information for further study on genetic diversity and conservation of Ephedraceae species.

**Figure 1.**
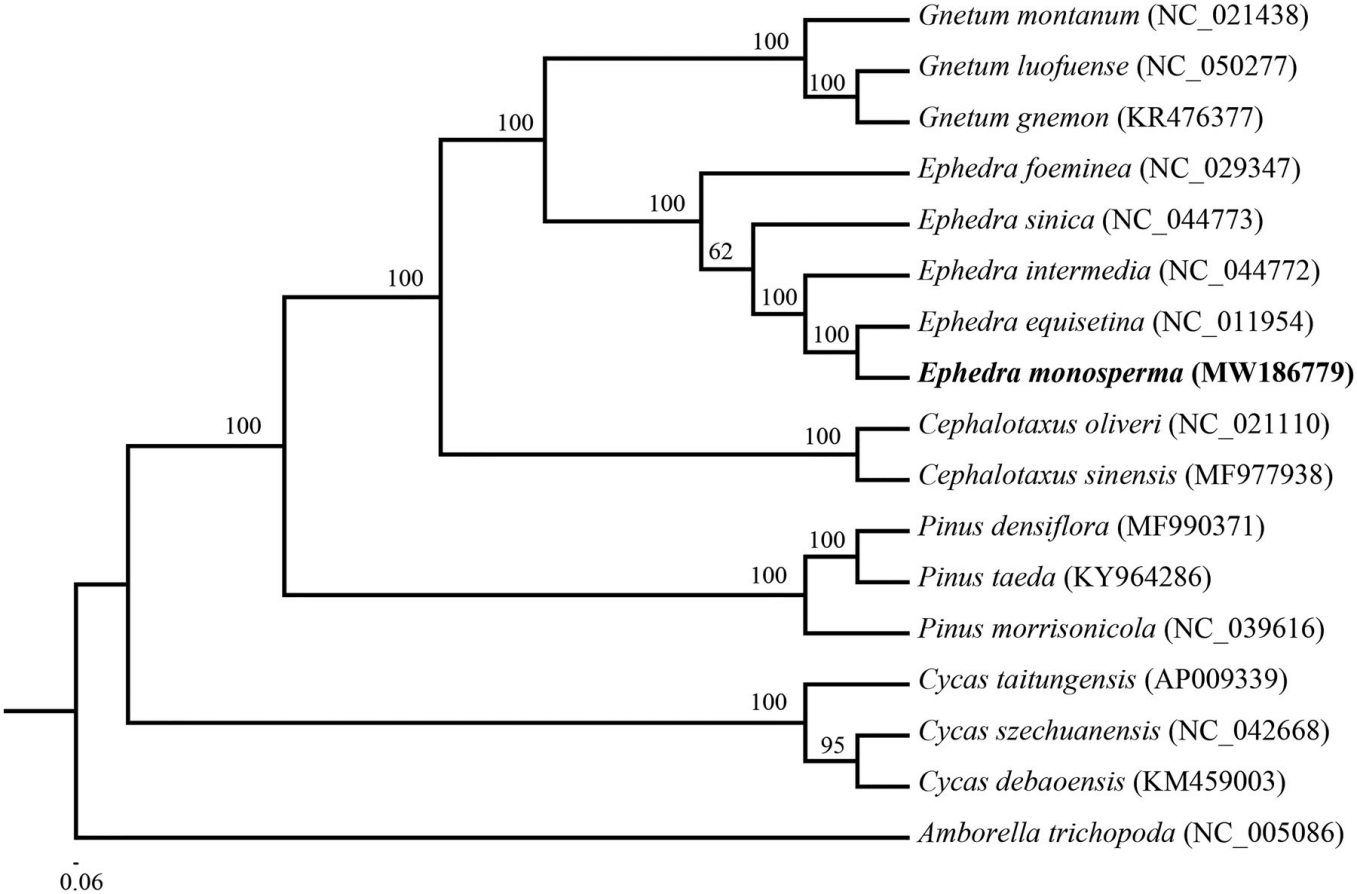
Maximum-likelihood phylogenetic tree for *E. monosperma* based on 17 complete chloroplast genome sequences. The number on each node indicates the bootstrap value.

## Data Availability

The genome sequence data that support the findings of this study are openly available in GenBank of NCBI at (https://www.ncbi.nlm.nih.gov/) under the accession no. MW186779. The associated BioProject, SRA, and Bio-Sample numbers are PRJNA681937, SRP295568, and SAMN16975003, SAMN16975004, respectively.
